# Prevalence of oral mucosal lesions in a brazilian military police population

**DOI:** 10.4317/jced.51934

**Published:** 2015-04-01

**Authors:** Viviani-Silva Araújo, Eliane-Lopes Godinho, Lucyana-Conceição Farias, Luciano Marques-Silva, Sérgio-Henrique-Sousa Santos, João-Felício Rodrigues-Neto, Raquel-Conceição Ferreira, Alfredo-Maurício-Batista De-Paula, Andréa-Maria-Eleutério-de Barros-Lima Martins, André-Luiz Sena-Guimarães

**Affiliations:** 1 MDS, Department of Dentistry, Universidade Estadual de Montes Claros, Montes Claros, Brazil; 2 PhD, Department of Dentistry, Universidade Estadual de Montes Claros, Montes Claros, Brazil; 3 DDS, Police Department of Minas Gerais State, Montes Claros, Brazil; 4 PhD, Department of Pharmacology, Universidade Federal de Minas Gerais,Belo Horizonte, Brazil; 5 PhD, Department of Medicine, Universidade Estadual de Montes Claros, Montes Claros, Brazil

## Abstract

**Background:**

Data obtained from oral health surveys are very important for identifying disease-susceptible groups and for developing dental care and prevention programs. So, the purpose of the current article was to investigate the prevalence of oral mucosa lesions (OMLs) in a population of Brazilian police.

**Material and Methods:**

Interviews and oral cavity examinations were performed on a sample of 395 police officers who were randomly selected by the calibrated researcher. The number of individuals was obtained by a sample calculation using the finite population correction. The diagnostic criteria were based on the WHO (1997) criteria and adapted to Brazilian surveys.

**Results:**

In total, 8.61% of the population presented some OML. Traumatic injuries and benign migratory glossitis (BMG) were the most prevalent lesions.

**Conclusions:**

The prevalence of potentially malignant disorders was lower than among the Brazilian population.The most prevalent lesion among the police officers was related to trauma. Patients dissatisfied with oral health had a higher risk of presenting OMLs.

** Key words:**Mouth disease, mouth mucosa, military personnel, public health, oral pathology, oral leukoplakia.

## Introduction

Occupational dentistry has become an important tool in the overall provision of occupational health care (1). Data obtained from oral health surveys are important for identifying groups susceptible to disease and developing dental care and prevention programs (2,3). However, few reports have examined the police population. Police activity involves frequent exposure to factors that may cause oral mucosal lesions (OMLs), such as armed confrontations, motor vehicle crashes and witnessing violent deaths (4,5). Additionally, environmental or local factors may affect police officers (6,7). It is important to highlight the similar prevalence rate of stress disorders between countries in policemen despite the marked difference in assessment methodology, local levels of violence, quality and duration of the training, and sociocultural factors (4). Considering these factors, the aim of the present study was to determine the frequency of OMLs in a Brazilian police population.

## Patient and Methods

A total of 803 police officers from Montes Claros, Minas Gerais, Brazil were studied. All patients had access to medical and dentistry services. The number of participants was defined by a sample calculation using the finite population correction. A total of 395 randomly selected police officers were included.

-Data Collection

The data were collected in personal interviews with the police officers. Examination of the oral cavity was performed after free and informed consent term (TFIC) was obtained. Data were collected from September 2008 to July 2009 by an experienced researcher. Oral clinical examination was based on the WHO (1997) guidelines and adapted to Brazilian surveys, as described previously (8). Following the oral exam, patients who had some type of OMLwere referred to the Oral and Maxillofacial Pathology and Stomatology Service of Universidade Estadual de Montes Claros-Brazil for diagnosis and treatment. The exclusion criteria were a lack of patient acceptance and TFIC signature.

-Sociodemographic Variables

The sociodemographic conditions assessed were age, marital status, sex, and position on the police force. The question, “How satisfied are you with your oral health?” was used to assess satisfaction with oral health. This variable was dichotomized as positive (very satisfied, satisfied, neither satisfied nor dissatisfied) and negative (dissatisfied, very dissatisfied). Tobacco and drinking habits were classified as described previously (9,10). Only patients who had never smoked were considered non-smokers. Ex-drinkers and ex-smokers were considered as such if they had abstained from any type of drinking and smoking for at least one year. Physical description of skin color was not used because it is a poor predictor of genomic ancestry in Brazil (11). Data collection was conducted in accordance with the ethical principles of the Helsinki Declaration, version 2002. The study was approved by the relevant institucional revisional boarding process number 1187/08.

-Statistical Analysis

Descriptive analyses of OMLs were performed. Additionally, a multivariate analysis was performed using binary logistic regression to construct a model of variables to provide a better explanation for the risk of OMLs. All analyses were assessed using SPSS 17.0 (SPSS Inc., Chicago), and statistical significance was set at *p*<0.05.

## Results

**Figure 1:**
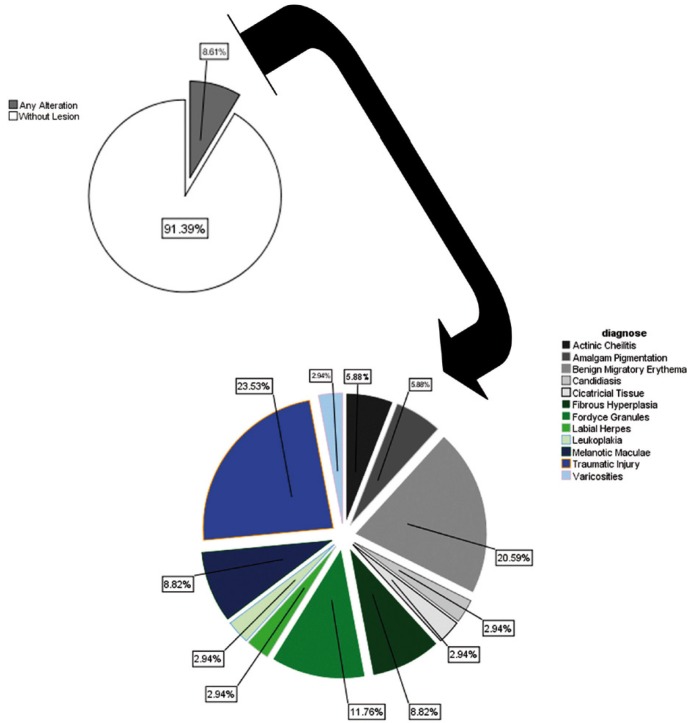
Distribution of oral mucosal lesions in the population.

A total of 8.61% of the studied population had some type of OML. The most common disorders were traumatic injuries (20.53%) and benign migratory glossitis (BMG) (20.59%) (Fig. 1). The prevalence of potential malignant disorders, such as oral leukoplakia and actinic cheilitis, was 0.3% and 0.5%, respectively ( Table 1).

**Table 1:**
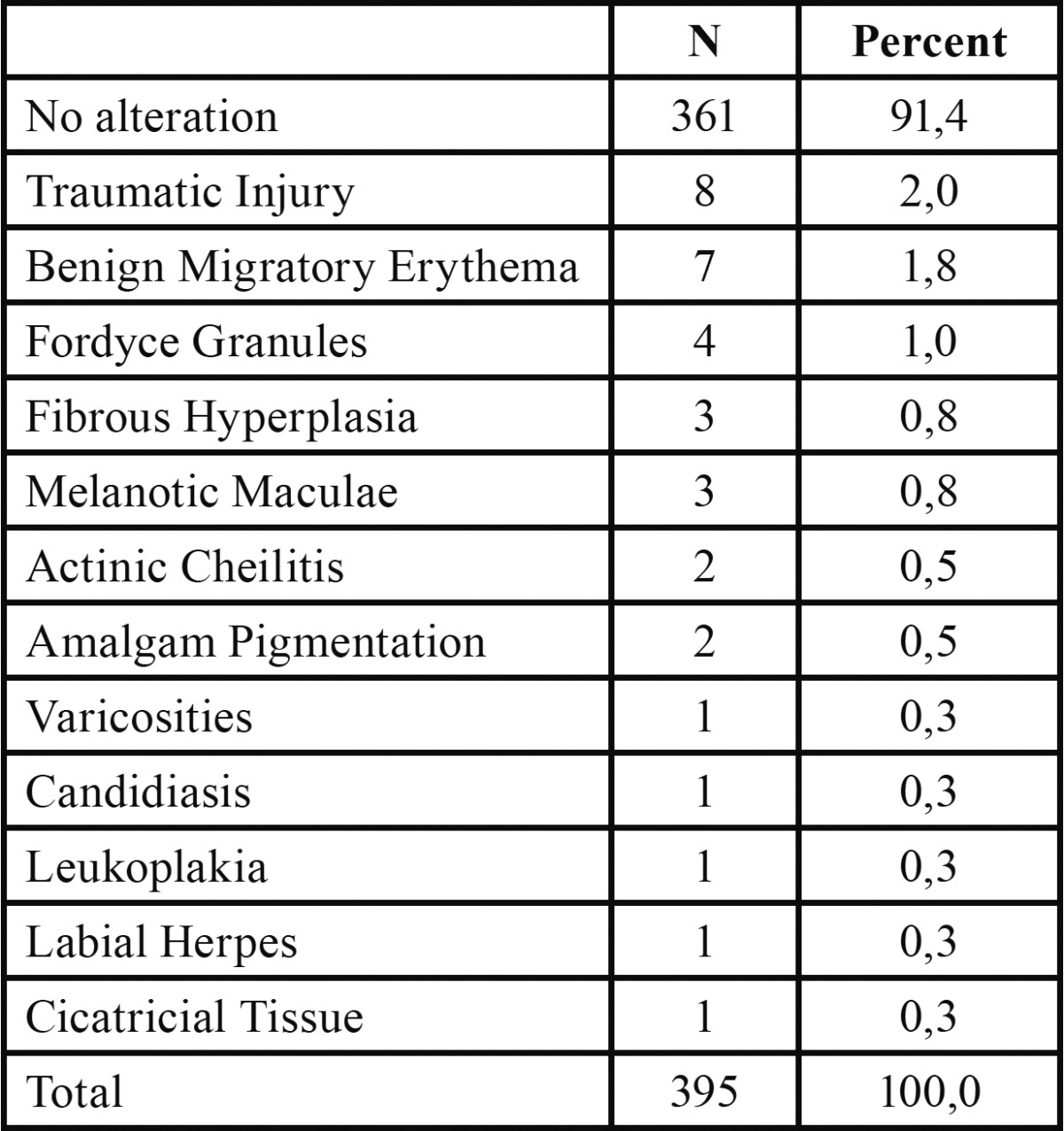
Prevalence of Oral Mucosal Lesions.

In multivariate analyses, dissatisfaction with oral health was an important factor associated with a higher risk of presenting OMLs ( Table 2).

**Table 2:**
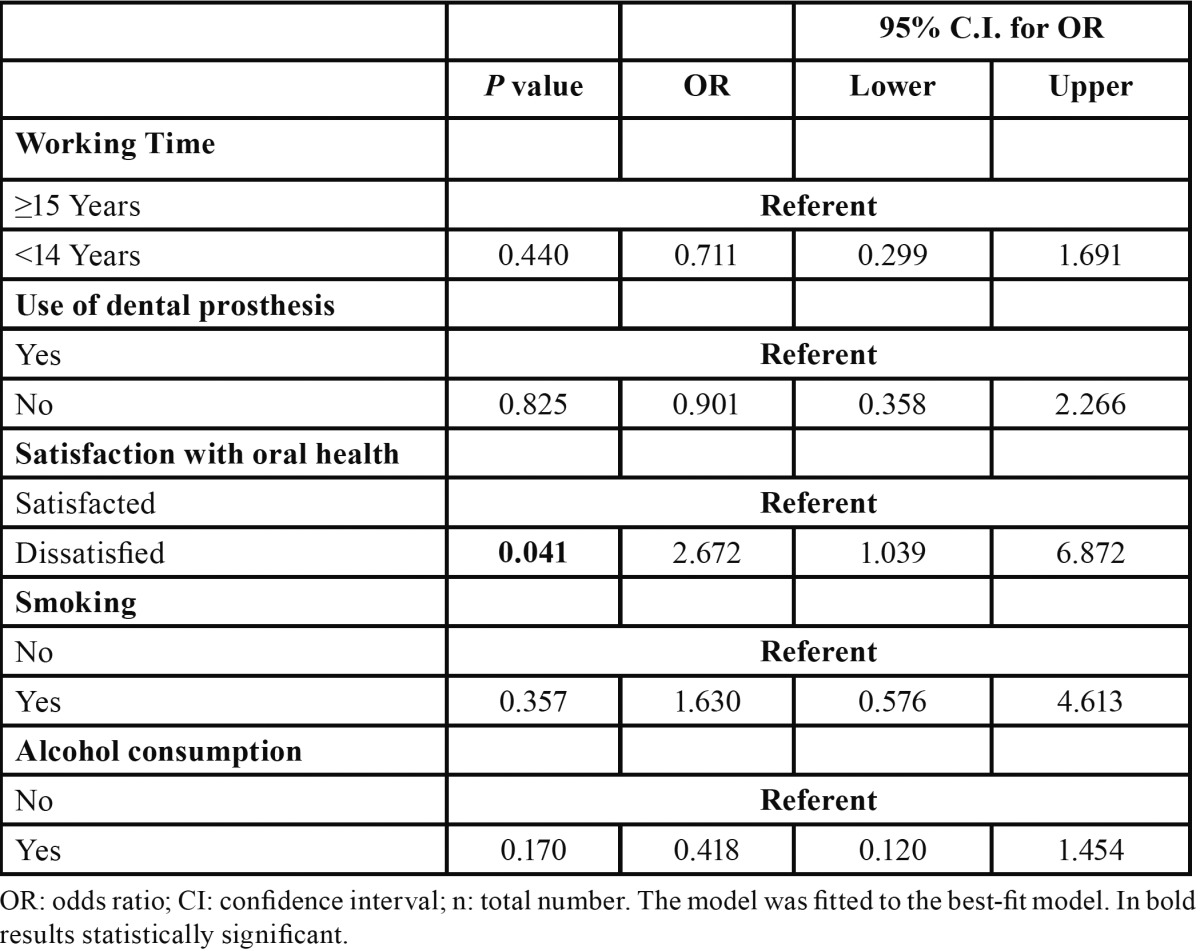
Risk to present Oral Mucosal Lesions.

## Discussion

Countries and companies are increasingly interested in occupational diseases, at least partially because of their financial consequences and the economic value of prevention (12). In contrast, little information on police has been available in the literature. To date, no study has attempted to investigate OMLs among a police population. The main role of the Minas Gerais Military Police (Polícia Militar de Minas Gerais) is crime prevention and combat. To achieve their goals, police officers are frequently exposed to potentially traumatic situations, such as armed confrontations, motor vehicle crashes and witnessing violent deaths (4). With regard to work activity, police officers can also be exposed to a plethora of factors associated with OMLs, such as UVB radiation (6,7), infections (13-17), medical treatment (18), local/chronic irritation and deleterious habits (9,10). Individual predispositions to OMLs, such as genetic and epigenetic factors, could also be important for the police worker (19-25).

In the current study, the prevalence of potentially malignant disorders was lower than among the general south Brazilian population (26). A lower incidence of actinic cheilitis was observed among the police population compared to agricultural workers (26). Our data could be explained by the population analyzed in the present study presenting a lower incidence of smoking (20.2%) in comparison to previous studies (45.27%) (27). This fact could be a consequence of frequent preventive and curative programs provided to police officers. It is difficult to compare Brazilian studies on oral health due to social differences (28) among study populations and the genetic diversity of the continental country population (11). It was observed that BMG represents 20.59% of all OMLs, with an incidence of 1.8%. Some reports in the literature indicate that BMG may be related to hormonal disturbances (29), psychological issues (30) and diabetes mellitus (31). However, the etiology of BMG remains unknown (32). Recently, we observed a possible association between genetic factors and BMG in a general population of the same state (21). In this context, future studies evaluating genetic predisposition to BMG would be useful to understand Brazilian variability. Traumatic injuries were the most prevalent findings observed in the current study and are directly associated with police activity. Finally, patients who were unsatisfied with oral health had a higher risk of presenting OMLs.

In conclusion, the most prevalent oral health issue among police officers was related to trauma. Patients who were dissatisfied with their oral health had a higher risk of presenting OMLs. Police position was associated with smoking and the need for a dental prosthesis.

## References

[1] Feaver GP (1988). Occupational dentistry: a review of 100 years of dental care in the workplace. J Soc Occup Med.

[2] Martins AM, Barreto SM, Pordeus IA (2008). [Factors associated to self perceived need of dental care among Brazilian elderly]. Rev Saude Publica.

[3] Martins AM, Barreto SM, Silveira MF, Santa-Rosa TT, Pereira RD (2010). Self-perceived oral health among Brazilian elderly individuals. Rev Saude Publica.

[4] Maia DB, Marmar CR, Metzler T, Nóbrega A, Berger W, Mendlowicz MV (2007). Post-traumatic stress symptoms in an elite unit of Brazilian police officers: prevalence and impact on psychosocial functioning and on physical and mental health. J Affect Disord.

[5] Carlier IV, Lamberts RD, Gersons BP (2000). The dimensionality of trauma: a multidimensional scaling comparison of police officers with and without posttraumatic stress disorder. Psychiatry Res.

[6] Souza LR, Fonseca-Silva T, Pereira CS, Santos EP, Lima LC, Carvalho H A (2011). Immunohistochemical analysis of p53, APE1, hMSH2 and ERCC1 proteins in actinic cheilitis and lip squamous cell carcinoma. Histopathology.

[7] Souza LR, Fonseca-Silva T, Santos CC, Oliveira MV, Corrêa-Oliveira R, Guimarães AL (2010). Association of mast cell, eosinophil leucocyte and microvessel densities in actinic cheilitis and lip squamous cell carcinoma. Histopathology.

[8] Mendes DC, Poswar Fde O, de Oliveira MV, Haikal DS, da Silveira MF, Martins AM (2012). Analysis of socio-demographic and systemic health factors and the normative conditions of oral health care in a population of the Brazilian elderly. Gerodontology.

[9] Farias LC, Fraga CA, De Oliveira MV, Silva TF, Marques-Silva L, Moreira PR (2010). Effect of age on the association between p16CDKN2A methylation and DNMT3B polymorphism in head and neck carcinoma and patient survival. Int J Oncol.

[10] De Paula AM, Souza LR, Farias LC, Corrêa GT, Fraga CA, Eleutério NB (2009). Analysis of 724 cases of primary head and neck squamous cell carcinoma (HNSCC) with a focus on young patients and p53 immunolocalization. Oral Oncol.

[11] Parra FC, Amado RC, Lambertucci JR, Rocha J, Antunes CM, Pena SD (2003). Color and genomic ancestry in Brazilians. Proc Natl Acad Sci U S A.

[12] Frazão P, Marques D (2009). Effectiveness of a community health worker program on oral health promotion. Rev Saude Publica.

[13] Correia-Silva Jde F, Victória JM, Guimarães AL, Salomão UE, de Abreu MH, Bittencourt H (2007). Cytomegalovirus shedding in the oral cavity of allogeneic haematopoietic stem cell transplant patients. Oral Dis.

[14] da Silva LM, Guimarães AL, Victória JM, Gomes CC, Gomez RS (2005). Herpes simplex virus type 1 shedding in the oral cavity of seropositive patients. Oral Dis.

[15] Guimaraes AL, Gomes CC, da Silva LM, Correia-Silva Jde F, Victoria JM, Gomez RS (2009). Association between oral HSV-1 and survival in allogeneic hematopoietic stem cell transplanted patients. Med Oral Patol Oral Cir Bucal.

[16] Marques-Silva L, Castro WH, Gomez EL, Guimarães AL, Silva MS, Gomez RS (2007). The impact of dental surgery on HSV-1 reactivation in the oral mucosa of seropositive patients. J Oral Maxillofac Surg.

[17] Victória JM, Guimarães AL, da Silva LM, Kalapothakis E, Gomez RS (2005). Polymerase chain reaction for identification of herpes simplex virus (HSV-1), cytomegalovirus (CMV) and human herpes virus-type 6 (HHV-6) in oral swabs. Microbiol Res.

[18] Gomez RS, Pimenta FJ, Guimarães AL, Souza LN, Salomão UE, de Almeida HC (2004). Effect of bone marrow transplantation on the immunolocalization of p53, hMSH2, and hMLH1 proteins on oral mucosa. Oral Dis.

[19] Moreira PR, Guimarães MM, Guimarães AL, Diniz MG, Gomes CC, Brito JA (2009). Methylation of P16, P21, P27, RB1 and P53 genes in odontogenic keratocysts. J Oral Pathol Med.

[20] Gomes CC, Drummond SN, Guimarães AL, Andrade CI, Mesquita RA, Gomez RS (2008). P21/ WAF1 and cyclin D1 variants and oral squamous cell carcinoma. J Oral Pathol Med.

[21] Guimarães AL, Correia-Silva Jde F, Diniz MG, Xavier GM, Horta MC, Gomez RS (2007). Investigation of functional gene polymorphisms: IL-1B, IL-6 and TNFA in benign migratory glossitis in Brazilian individuals. J Oral Pathol Med.

[22] Guimarães AL, Correia-Silva Jde F, Sá AR, Victória JM, Diniz MG, Costa FdeO (2007). Investigation of functional gene polymorphisms IL-1beta, IL-6, IL-10 and TNF-alpha in individuals with recurrent aphthous stomatitis. Arch Oral Biol.

[23] Guimarães AL, de Sá AR, Victória JM, Correia-Silva JF, Pessoa PS, Diniz MG (2006). Association of interleukin-1beta polymorphism with recurrent aphthous stomatitis in Brazilian individuals. Oral Dis.

[24] Guimarães AL, de Sá AR, Victoria JM, de Fátima Correia-Silva J, Gomez MV, Gomez RS (2006). Interleukin-1beta and serotonin transporter gene polymorphisms in burning mouth syndrome patients. J Pain.

[25] Xavier GM, de Sá AR, Guimarães AL, da Silva TA, Gomez RS (2007). Investigation of functional gene polymorphisms interleukin-1beta, interleukin-6, interleukin-10 and tumor necrosis factor in individuals with oral lichen planus. J Oral Pathol Med.

[26] Junqueira JL, Bönecker M, Furuse C, Morais Pde C, Flório FM, Cury PR (2011). Actinic cheilitis among agricultural workers in Campinas, Brazil. Community Dent Health.

[27] Carrard V, Haas A, Rados P, Filho M, Oppermann R, Albandar J (2011). Prevalence and risk indicators of oral mucosal lesions in an urban population from South Brazil. Oral Dis.

[28] Victora CG, Barreto ML, do Carmo Leal M, Monteiro CA, Schmidt MI, Paim J (2011). Health conditions and health-policy innovations in Brazil: the way forward. Lancet.

[29] Waltimo J (1991). Geographic tongue during a year of oral contraceptive cycles. Br Dent J.

[30] Redman RS, Vance FL, Gorlin RJ, Peagler FD, Meskin LH (1966). Psychological component in the etiology of geographic tongue. J Dent Res.

[31] Wysocki GP, Daley TD (1987). Benign migratory glossitis in patients with juvenile diabetes. Oral Surg Oral Med Oral Pathol.

[32] Assimakopoulos D, Patrikakos G, Fotika C, Elisaf M (2002). Benign migratory glossitis or geographic tongue: an enigmatic oral lesion. Am J Med.

